# Conditional Deletion of TAK1 in T Cells Reveals a Pivotal Role of TCRαβ^+^ Intraepithelial Lymphocytes in Preventing Lymphopenia-Associated Colitis

**DOI:** 10.1371/journal.pone.0128761

**Published:** 2015-07-01

**Authors:** Hideki Sanjo, Shigeo Tokumaru, Shizuo Akira, Shinsuke Taki

**Affiliations:** 1 Department of Molecular and Cellular Immunology, Shinshu University School of Medicine, Nagano, Japan; 2 Laboratory of Host Defense, Immunology Frontier Research Center, Osaka University, Osaka, Japan; Indiana University School of Medicine, UNITED STATES

## Abstract

The kinase TAK is required for the development of conventional and regulatory T cells. We previously reported that mice with conditional deletion of TAK1 in T cells (Lck-cre:TAK1^fl/fl^ mice) exhibited severe T lymphopenia, and were nevertheless predisposed to spontaneous colitis with unknown etiology. Here we focused on the immunopathological mechanism in colitic Lck-cre:TAK1^fl/fl^ mice. We found that ‘leaky’ CD4^+^ T cells retaining TAK1 acquired inflammatory phenotypes that contribute to disease onset in Lck-cre:TAK1^fl/fl^ mice. Furthermore, the gut microbiota-triggered signaling was also a key event leading to the pathogenesis. We discovered that Lck-cre:TAK1^fl/fl^ mice were almost completely devoid of TCRαβ^+^CD8α^+^ intestinal intraepithelial lymphocytes (IELs) and this was largely due to the developmental defect of the thymic precursors by TAK1 deficiency. Remarkably, transfer of TCRαβ^+^CD8α^+^ IELs from wild-type mice ameliorated colitis in Lck-cre:TAK1^fl/fl^ mice. Taken together, our current study highlighted the emerging role of TAK1 in configuring the gut-specialized T cell subset, which regulates mucosal homeostasis under lymphopenic conditions.

## Introduction

Inflammatory bowel disease (IBD) is a chronic inflammatory disorder. The two main types of IBD, Crohn’s disease and ulcerative colitis, both of which are caused by the breakdown of immune homeostasis in the gut, manifest persistent intestinal inflammation associated with both remission and relapse. The causes of disease onset are complex but environmental, host genetic and commensal microbial factors have been implicated in IBD [1,2]. As recently pointed out, commensal bacteria themselves deeply affect gut immune homeostasis via their own products, underscoring the importance of a mutual relationship between host and commensal microbiota [3–7].

In order to study IBD, a lot of researchers have addressed using some experimental animal models. For instance, in mice treatment with dextran sulfate sodium or 2,4,6-trinitrobenzene sulfonic acid reveals acute colitis involves loss of the epithelial barrier function of intestine [8]. In contrast, chronic colitis occurs when naive CD4^+^ T cells are transferred into T cell-deficient mice [9]. Moreover it was reported that chronic IBD-like diseases were observed in mice deficient in some cytokines, transcription factors or TCR components [10–14].

Intraepithelial lymphocytes (IELs) are comprised of heterogeneous cell populations that are nestled among epithelial cells in mucosal linings. In particular, intestinal IELs are believed to contribute to the maintenance of the mucosal barrier function, along with enterocytes, by preventing pathogen penetration [15–17]. These findings among many others point towards the importance of IELs in colitis studies [18–22]; however, the regulatory mechanism exerted by IELs to suppress inflammation in the gastrointestinal tract remains poorly defined.

TAK1, a serine/threonine kinase belonging to MAPKKK family, plays a central role in regulating cell survival, proliferation and differentiation in vertebrates and invertebrates [23]. Conditional deletion of TAK1 specifically in T lymphocytes results in the inability of T cells to propagate antigen receptor and some cytokine signals [24–26]. We also found that T cell-specific TAK1 knockout mice (Lck-cre:TAK1^fl/fl^ mice) showed late-onset spontaneous colitis ~3 months after birth despite severe T lymphopenia [25]. However, it is not well understood why and how the symptom of IBD occurs in those mice.

Here we characterized the pathological processes in colitic Lck-cre:TAK1^fl/fl^ mice, revealing a dominant population of CD4^+^ T cells, with incomplete Cre-mediated deletion of the TAK1 gene, populated the mesenteric lymph nodes (mLNs) and colonic lamina propria (cLP) of the mice. These cells, without TAK1 deleted, exhibited a colitogenic cytokine profile. In the periphery, colitic Lck-cre:TAK1^fl/fl^ mice possessed considerable numbers of Foxp3^+^ regulatory T cells, which also retain TAK1; however, suppressive activity that regulatory T cells have in themselves was significantly decreased. The gut microbiota-triggered signaling also contributed to the pathogenesis of the mice. Intriguingly, in both small and large intestine of Lck-cre:TAK1^fl/fl^ mice, TCRαβ^+^CD8αα^+^ IEL subset was almost completely absent even in older animals, most likely due to the lack of TAK1-dependent TCR signaling in the thymic precursors for IELs. Transfer of TCRαβ^+^CD8α^+^ IELs but not any other T cell subsets such as conventional CD8^+^ T or NKT cells ameliorated colitis in Lck-cre:TAK1^fl/fl^ mice. Collectively, our data reveal the emerging role of TAK1 in configuring the gut-specialized T cell subset; an avenue that may be essential for immune homeostasis in the gut.

## Materials and Methods

### Ethics statement

All studies were approved by the Shinshu University Animal Care Committee (Approval Number: 260018) and all the experiments using animal were performed according to the guideline presented by the committee.

### Mice

Lck-cre:TAK1^fl/fl^ mice [25], used as LTAC mice, TAK1^fl/fl^ littermate control mice, as wild-type (WT) mice and MyD88^–/–^ mice, all of which are C57BL/6 background (CD45.2^+^), and C57BL/6 (CD45.1^+^) mice were maintained at the Shinshu University animal facilities under specific pathogen-free conditions.

### Cell isolation

Mice were euthanized by intraperitoneal injection of a large excess of pentobarbital sodium. Isolated colons were cut, opened longitudinally and washed with excess PBS to remove stools and mucus, followed by incubation for 30 min at 37°C with vigorous shaking in Ca^2+^- and Mg^2+^-free Hanks balanced salt solution supplemented with 5 mM EDTA and 1 mM DTT to remove epithelial layer. Cells released from colons were subjected to 40% / 70% Percoll gradient centrifugation and the cells containing IELs in middle layer were harvested and used for experiments. To isolate lamina propria cells, the remaining colons after removing epithelial layer were chopped into little pieces with scalpel and digested for 60 min at 37°C with gentle shaking in RPMI1640 medium supplemented with 5% FBS, 0.5 mg/ml Collagenase IV (SIGMA) and 50 U/ml DNase I (Wako). Cells were subjected to 40% / 70% Percoll gradient centrifugation and the cells in middle layer were harvested and used for experiments.

### Antibodies, reagents, flow cytometry and cell sorting

Fluorochrome- or biotin-conjugated antibodies and reagents used in this research are as follows: TCRβ (H57-597), CD3 (145-2C11), CD11b (M1/70), CD45.2 (104), CD45.1 (A20), CD62L (MEL-14), TER-119 (TER-119), F4/80 (BM8), CCR9 (eBioCW-1.2), Foxp3 (FJK-16s), IFNγ (XMG1.2) and Fixable Viability Dye eFluor 780 all from eBioscience; CD4 (RM4-5), CD5 (53–7.3), CD8α (53–6.7), CD8β (53–5.8), CD11c (HL3), CD44 (IM7), B220 (RA3-6B2), CD122 (TM-β1), TCRγδ (GL3), mIgG1 (A85-1), I-Ab (AF6-120.1), Dimer X mouse CD1d:Ig fusion protein and iMAG streptavidin all from BD Bioscience; CD11c (N418), CD25 (PC61), CD103 (2E7), NK1.1 (PK136), α4β7 (DATK32), IL-17A (TC11-18H10.1) and Brilliant Violet 421-conjugated streptavidin all from BioLegend. α-galactosylceramide (α-GalCer) was kindly provided by Kyowa Hakko Kirin. Anti-CD16/CD32 antibody (2.4G2) was prepared from hybridoma. Foxp3 Transcription Factor Staining Buffer Set (eBioscience) was used for intracellular staining of Foxp3. For intracellular cytokine staining, cells were cultured with 50 ng/ml phorbol 12-myristate 13-acetate (PMA) (Wako) plus 500 ng/ml ionomycin (Wako) for 5 hours in the presence of GolgiStop (BD Bioscience). After staining with antibodies to cell surface molecules, cells were fixed and permeabilized in Cytofix/Cytoperm buffer (BD Bioscience), followed by intracellular cytokine staining in Perm/Wash buffer (BD Bioscience). Stained cells were analyzed with FACS Canto II flow cytometer (BD Bioscience) or sorted with FACS Aria III cell sorter (BD Bioscience). Flow cytometry data were analyzed with Kaluza software (Beckman Coulter).

### Real time PCR

Total RNAs were prepared from colons or mLNs, followed by cDNA synthesis with reverse transcriptase. Real time PCR was performed using SYBR Premix Ex Taq II (TaKaRa) and Thermal Cycler Dice Real Time System (TaKaRa) according to manufacture’s instruction. Each data was normalized by values of GAPDH used as an internal control. Primers used in this research are as follows: IL-1β forward, 5’-TGAGCTGAAAGCTCTCCACC-3’ and reverse, 5’-CCAAGGCCACAGGTATTTTG-3’; IL-6 forward, 5’-CTTCAACC AAGAGGTAAAAG-3’ and reverse, 5’-GATGGTCTTGGTCCTTAGCC-3’; IL-12 p40 forward, 5’-GGGTGTAACCAGAAAGGTGC-3’ and reverse, 5’-TGCCCACT TGCTGCATGAGG-3’; TNFα forward, 5’-AGCCCACGTCGTAGCAAACC-3’ and reverse, 5’-CTGGCACCACTAGTTGGTTG-3’; IFNγ forward, 5’-TTCTTCAGCAA CAGCAAGGC-3’ and reverse, 5’-GCTGGTGGACCACTCGGATG-3’; IL-17A forward, 5’-TGGACTCTCCACCGCAATGA-3’ and reverse, 5’-CTCTTGCTGGA TGAGAACAG-3’; IL-22 forward, 5’-GGAGACAGTGAAAAAGCTTG-3’ and reverse, 5’-AGCTTCTTCTCGCTCAGACG-3’; IL-23 p19 forward, 5’-CAACTCCT CCAGCCAGAGGA-3’ and reverse, 5’-GAGGCTTCGAAGGATCTTGG-3’; GM-CSF forward, 5’-CCCGCCTGAAGATATTCGAG-3’ and reverse, 5’-TTCACA GTCCGTTTCCGGAG-3’; IL-10 forward, 5’-AGAGCAAGGCAGTGGAGCAG-3’ and reverse, 5’-CATCATGTATGCTTCTATGC-3’; TGFβ forward, 5’-GACTTTAG GAAGGACCTGGG-3’ and reverse, 5’-GGGCAAGGACCTTGCTGTAC-3’; Reg3β forward, 5’-GGAATGGAGTAACAATGACG-3’ and reverse, 5’-GGGCA ACTTCACCTCACATG-3’; Reg3γ forward, 5’-CCATCTTCACGTAGCAGCTG-3’ and reverse 5’-GGGCAACTTCACCTCACATG-3’; Muc-2 forward, 5’-CCTACAA TGGCTGCACCAAG-3’ and reverse 5’-CTTCGGACACTGGTCTTCTC-3’; RALDH1 forward, 5’-ATGGTTTAGCAGCAGGACTCTTC-3’ and reverse, 5’-CCA GACATCTTGAATCCACCGAA-3’; RALDH2 forward, 5’-GACTTGTAGCAGCT GTCTTCACT-3’ and reverse, 5’-TCACCCATTTCTCTCCCATTTCC-3’.

### Cell culture

Thymocytes after depletion of CD4^+^, CD8^+^ and NK1.1^+^ cells were cultured with plate-bound anti-CD3 antibody (10 μg/ml) in the absence or presence of IL-2 (500 U/ml; Ajinomoto) for 3 days, followed by flow cytometry analysis. For *in vitro* suppression assay, CD45.1^+^CD4^+^CD25^–^ T cells were labeled with 5 μM CFSE and cultured with soluble anti-CD3 antibody (5 μg/ml) and T cell-depleted splenocytes in the absence or presence of CD4^+^CD25^+^ regulatory T cells for 3 days. Fluorescence intensity of CFSE was measured by flow cytometry.

### Adoptive T cell transfer

Spleens from CD45.1^+^ C57BL/6 mice were isolated and splenocytes after depletion of non-T cell populations were stained with antibodies against TCRβ and CD8α or TCRβ and NK1.1. TCRβ^+^CD8α^+^ cells as CD8^+^ T cells or TCRβ^+^NK1.1^+^ cells as NKT cells were sorted. IELs purified from small and large intestines of CD45.1^+^ C57BL/6 mice were stained with antibodies against TCRβ, TCRγδ and CD8α and TCRβ^+^CD8α^+^ IELs were sorted. Each T cell subset with > 95% purity was intravenously transferred into 6- to 8-week-old mice. Cell number used for transfer is as follows: CD8^+^ T, 2 x 10^6^ cells / mouse; NKT, 5 x 10^5^ cells / mouse; TCRβ^+^CD8α^+^ IELs, 5 x 10^5^ cells / mouse. Eight weeks after transfer, the mice were used for analysis.

### *In vivo* permeability assay

*In vivo* permeability assay to assess intestinal barrier function was performed using an FITC-labeled dextran method. Briefly, 8- to 12-week-old mice fasted for 12 hours were rectally administered with FITC-labeled dextran (2 mg/10 g body weight, MW 4000, SIGMA). Blood was collected after 3 hours and serum fluorescence intensity was measured. FITC-dextran concentrations were calculated from standard curves generated by serial dilution of FITC-dextran.

### Bacterial culture

Total liver cell suspensions prepared from more than 12-week-old mice were cultured in sorbitol macconkey agar plates (Kyokuto) for 24 hours at 37°C.

### Antibiotics treatment

Six to 8-week-old mice were fed drinking water containing antibiotics (ampicillin: 1 g/liter, vancomycin: 0.5 g/liter, neomycin: 1 g/liter, metronidazole: 1 g/liter) for 8 weeks to deplete gut commensal bacteria.

### Histopathology

Sections from formalin-fixed and paraffin-embedded colons were subjected to hematoxylin-eosin (HE) staining. Stained sections were blindly scored for severity of colitis according to the following assessment previously reported with small modification [27]: inflammation severity (0, none; 1, mild; 2, moderate; 3, severe), inflammatory extent (0, none; 1, mucosa, 2, mucosa and submucosa; 3, transmural), abnormal crypt morphology (0, none; 1, moderate; 2. Severe) and goblet cell loss (0, none; 1, moderate; 2, severe). Total colitis score was derived from the sum of the above subscores (maximum score: 10).

### Statistics

Statistical analysis was performed with Graph Pad Prism software using unpaired two-tailed *t* test, Mann-Whitney test or one-way analysis of variance (ANOVA) Bonferroni’s multiple comparison test. Statistical significance was assumed at *P* values less than 0.05.

## Results

### Development of spontaneous colitis in Lck-cre:TAK1^fl/fl^ mice

**Fig 1:**
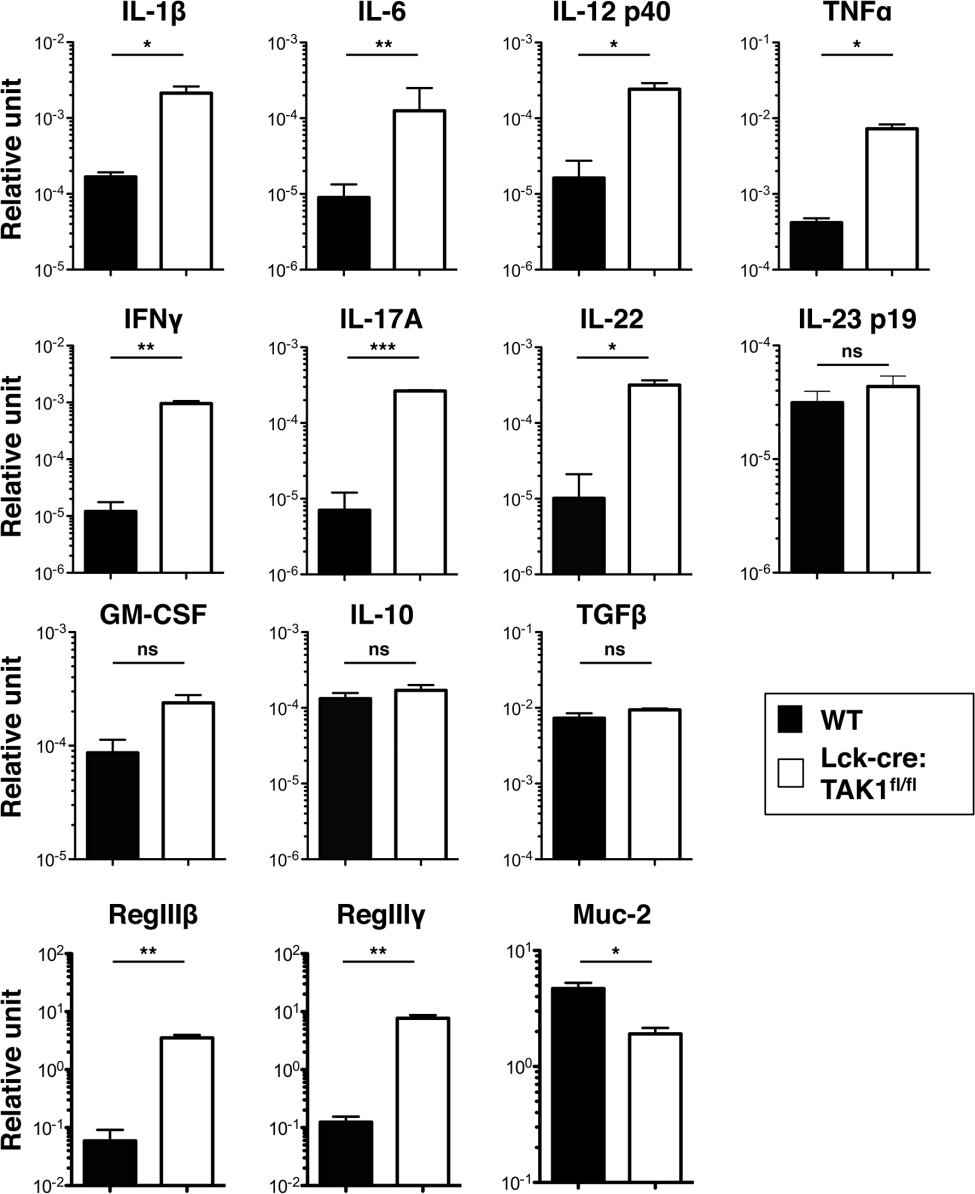
Altered expression profile of cytokines, anti-microbial peptides and an intestinal mucus in the colon of Lck-cre:TAK1^fl/fl^ mice. Total RNAs extracted from proximal colons of 12- to 16-week-old wild-type (WT) and Lck-cre:TAK1^**fl/fl**^ mice were used for real time PCR analysis. Data are representative of three independent experiments (mean ± standard deviation (s.d.)). Unpaired *t* tests were performed. Statistical significance was indicated by *P < 0.05, **P < 0.01, ***P < 0.001; ns, not significant.

Consistent with our previous study [25], real time PCR analysis using RNA prepared from the colon showed conspicuously increased mRNA levels of proinflammatory cytokines and RegIIIβ/γ proteins, known as antimicrobial peptides produced from the intestinal epithelium in Lck-cre:TAK1^fl/fl^ mice (Fig 1). A significantly decreased expression of Muc-2, a gut-specific mucus, was also observed in these mice (Fig 1). There were no differences in expression levels of the two anti-inflammatory cytokines IL-10 and TGFβ between wild-type and Lck-cre:TAK1^fl/fl^ mice (Fig 1). Taken together, these observations, particularly the elevated expression of the helper T cells (Th)-related cytokines IFNγ and IL-17, suggested the involvement of Th cells in colitis in Lck-cre:TAK1^fl/fl^ mice.

### Accumulation of T cells retaining TAK1 in colonic lamina propria of LTAC mice

**Fig 2:**
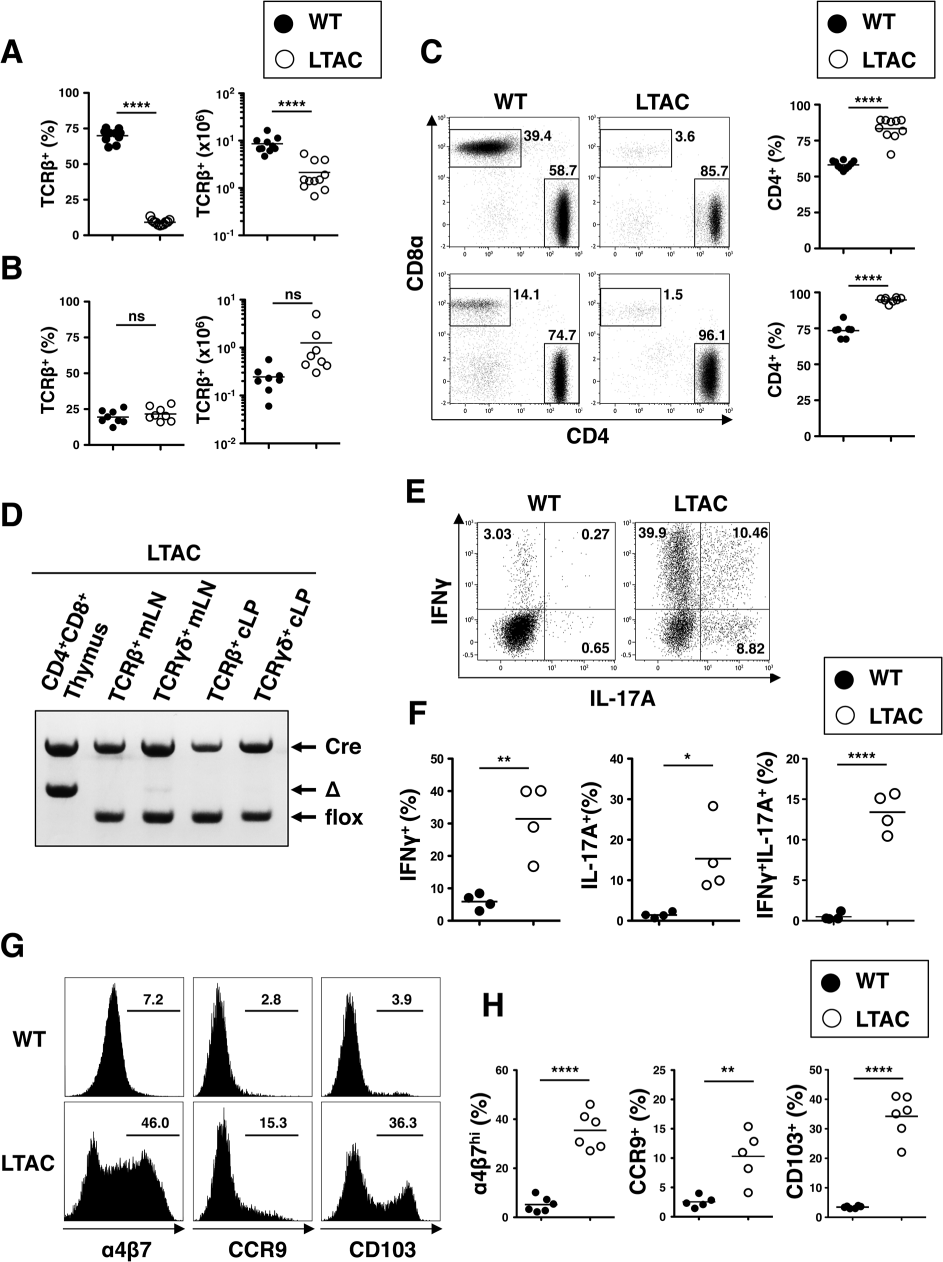
Characterization of aberrant T cells in LTAC mice. (A and B) Frequencies (left) and absolute numbers (right) of TCRβ^**+**^ cells of CD45^**+**^ cells from mLNs (A) or cLP (B) of WT (n = 9–10, filled circle) and LTAC mice (n = 9–11, open circle) determined by flow cytometry analysis. Horizontal bars represent mean. (C) (Left and middle plots) Flow cytometry of CD4^**+**^ and CD8α^**+**^ cell subsets in TCRβ^**+**^ cells. (Right panels) Frequencies of CD4^**+**^ cell subset in TCRβ^**+**^ cells. The plots are representative of at least three independent experiments. mLNs (upper) or cLP (lower) of WT (n = 7–10, filled circle) and LTAC mice (n = 8–10, open circle), determined by flow cytometry analysis. Horizontal bars represent mean. (D) Genomic DNAs from the sorted T cells in each tissue indicated were subjected to PCR amplification to detect flox (= TAK1 WT) or Δ (= TAK1 KO) allele in the TAK1 genomic locus. The DNA size difference between the flox and Δ bands is shown. Specific DNAs for Cre, loaded in each well, were also shown to prove its existence in the genome. (E) Flow cytometry of intracellular cytokines in TCRβ^**+**^CD4^**+**^ cells from cLP after culture with PMA + Ionomycin for 5 hours in the presence of GolgiStop. The plots are representative of four independent experiments. (F) Frequencies of each subset of cytokine-producing cells in TCRβ^**+**^CD4^**+**^ cells in cLP of WT (n = 4, filled circle) and LTAC mice (n = 4, open circle), calculated by flow cytometry analysis from (E). Horizontal bars represent mean. (G) Flow cytometry of gut homing receptors by mLN TCRβ^**+**^CD4^**+**^ cells. The histograms are representative of at least three independent experiments. (H) Frequencies of α4β7^**hi**^, CCR9^**+**^ and CD103^**+**^ cells of TCRβ^**+**^CD4^**+**^ cells in the mLNs of WT (n ≧ 5, filled circle) and LTAC mice (n ≧ 5, open circle), determined by flow cytometry analysis from (H). Horizontal bars represent mean. In (A), (B), (C), (F) and (H), unpaired *t* tests were performed. Statistical significance was indicated by *P < 0.05, **P < 0.01, ****P < 0.0001; ns, not significant.

A characteristic feature regarding cytokine profile of colitic Lck-cre:TAK1^fl/fl^ mice, herein referred to as LTAC (Lck-cre:TAK1^fl/fl^ conditional knockout-associated colitis) mice, prompted us to reevaluate LTAC mice as a potentially unique mouse model for IBD. We first focused on sites of inflammation. Consistent with our previous report [25], LTAC mice exhibited a drastic reduction in the frequency and absolute number of TCRβ^+^ T cells in the mLNs when compared with wild-type mice (Fig 2A). Surprisingly, the cLP contained TCRβ^+^ T cells almost at the same frequency in both wild-type and LTAC mice (Fig 2B). We noted that some but not all LTAC mice had up to 10 times more T cells in the cLP than wild-type mice (Fig 2B). We further observed that the vast majority of TCRαβ^+^ T cells in the mLNs and cLP of LTAC mice were CD4^+^ T cells compared with wild-type mice (Fig 2C).

We next questioned why unchanged frequency of TCRβ^+^ T cells could be detected in the cLP of LTAC mice in spite of the T lymphopenic phenotype in mLNs. To address this issue, we checked for TAK1 deletion efficiency in the T cells by PCR of genomic DNA. As expected from our previous report [25], CD4^+^CD8^+^ thymocytes sorted from the thymus of LTAC mice exhibited almost a complete deletion of the specific region flanked by loxP sites in the TAK1 genomic locus by the expression of Lck promoter-driven Cre protein. Surprisingly, such a deletion was scarcely detected in both TCRαβ^+^ and γδ^+^ T cell subsets sorted from the mLNs and cLP in LTAC mice (Fig 2D), indicating that these cells still have intact TAK1 proteins.

The fact that the elevated inflammatory cytokine expression such as IFNγ and IL-17A in the colon and accumulation of CD4^+^ T cells in cLP were observed in LTAC mice raised the question of whether these T cells turned out to gain pathogenic characters that would contribute to onset of colitis. To address this question, we analyzed cytokines produced from T cells. When CD4^+^ T cells isolated from cLP and mLNs were stimulated with PMA plus ionomycin, a more robust production of IFNγ and IL-17A was detected in the cells from LTAC mice than in those from wild-type mice (Fig 2E and 2F and S1 Fig). Conceivably, these T cells were equivalent to the colitogenic CD4^+^ T cells producing both IFNγ and IL-17A often observed in mice with IBD.

Given that CD4^+^ T cells activated in mLNs express gut homing receptors such as α4β7 integrin, CCR9 and CD103, also known as αE integrin, and gain access to the lamina propria, we speculated that accumulation of CD4^+^ T cells in the cLP of LTAC mice might be due to excess entry of cells expressing these homing receptors from the mLNs. To prove this hypothesis, we analyzed the expression pattern of these homing receptors on CD4^+^ T cells in the mLNs of LTAC mice. We observed that all the homing receptors were strongly expressed on CD4^+^ T cells from LTAC mice compared with those from wild-type mice (Fig 2G and 2H). Taken together, these data clearly demonstrated that the ‘leaky T cells’, which have the residual TAK1, acquired an inflammatory phenotype, and continuous access to the colon resulted ultimately in the pathogenesis of colitis in LTAC mice.

### Appearance of aberrant regulatory T cells with TAK1 in LTAC mice

**Fig 3:**
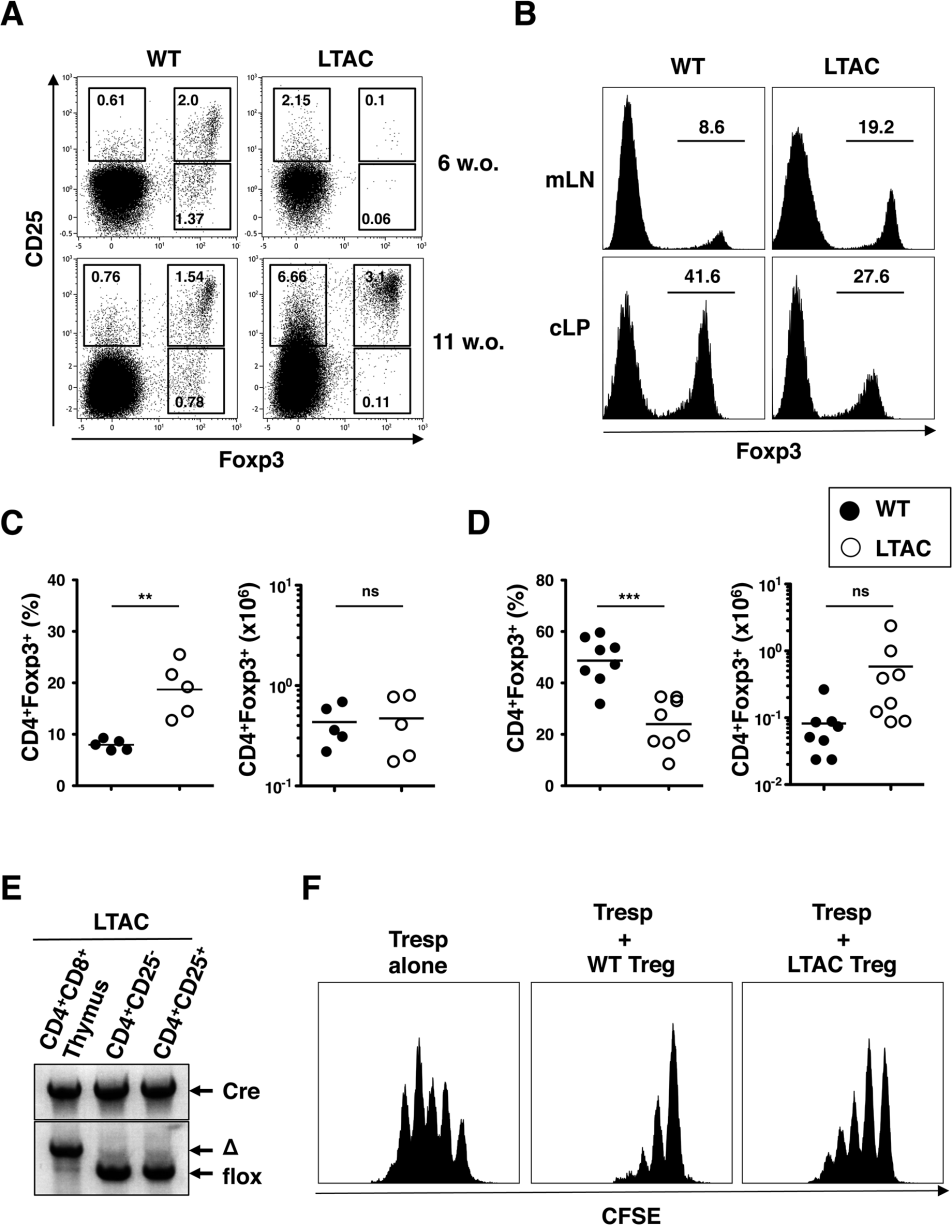
Regulatory T cell population in LTAC mice. (A) Flow cytometry analysis with thymocytes from 6-week-old (w.o.) or 11 w.o. WT and LTAC mice. Staining for CD25 and Foxp3 determined Treg in CD4 single positive cells. The plots are representative of three independent experiments. (B) Foxp3 expression in TCRβ^**+**^CD4^**+**^ cells in tissues indicated. The histograms are representative of at least five independent experiments. (C and D) Frequencies (left) and absolute numbers (right) of Foxp3^**+**^ cells of TCRβ^**+**^CD4^**+**^ cells from the mLNs (C) and cLP (D) of WT (n = 5–8, filled circle) and LTAC mice (n = 5–8, open circle), determined by flow cytometry analysis from (B). Horizontal bars represent mean. (E) Genomic DNAs from the sorted cells indicated, followed by PCR amplification to detect flox or Δ allele in TAK1 genomic locus like Fig 2C. (F) CFSE-labeled CD45.1^**+**^CD4^**+**^CD25^**–**^ T cells (Tresp) were cultured with soluble anti-CD3 antibody and T cell-depleted splenocytes in the absence (Tresp alone) or presence of CD4^**+**^CD25^**+**^ regulatory T cells (Treg) purified from wild-type or LTAC mice for 3 days (The ratio of Tresp to Treg is 2:1.). The histograms are representative of three independent experiments. In (C) and (D), unpaired *t* tests were performed. Statistical significance was indicated by **P < 0.01, ***P < 0.001; ns, not significant.

We previously reported that TAK1 deficiency in T cells resulted in an impaired generation of regulatory T cells [25] and in fact we confirmed a dramatic reduction in the frequency of CD25^+^Foxp3^+^ cells in CD4 single positive thymocytes of 6-week-old LTAC mice in which colitis does not appear (Fig 3A). Unexpectedly, however, we recognized regulatory T cells in peripheral tissues such as mLNs and cLP of more than 10-week-old LTAC mice as well as wild-type mice and its frequency was significantly higher and lower in the mLNs and cLP, respectively, of LTAC mice than those of wild-type mice (Fig 3B–3D), whilst absolute numbers of regulatory T cells were virtually unchanged in the mLNs and cLP of both types of mice (Fig 3C and 3D). When we checked regulatory T cells in the thymus of more than 10-week-old LTAC mice, we noticed that CD4^+^CD25^+^Foxp3^+^ regulatory T cells were as frequent as those in wild-type mice (Fig 3A). Having observed the failure of TAK1 deletion in the remaining CD4^+^ T cells in peripheral tissues of LTAC mice, it would be plausible that the regulatory T cells in LTAC mice still possess intact TAK1. Indeed, the cells sorted from mLNs of LTAC mice showed virtually no sign for deletion of the TAK1 genomic locus (Fig 3E), indicating that regulatory T cells we observed in LTAC mice have intact TAK1. When we performed *in vitro* suppression assay to investigate regulatory T cell function, intriguingly, regulatory T cells in LTAC mice showed less suppressive activity than that of wild-type regulatory T cells (Fig 3F). Taken together, these data suggested that failure of suppressive function of the leaky regulatory T cells facilitated the pathogenesis of colitis in LTAC mice.

### Defective barrier function and bacteria activated pathogen recognition receptors lead to colitis development in LTAC mice

**Fig 4:**
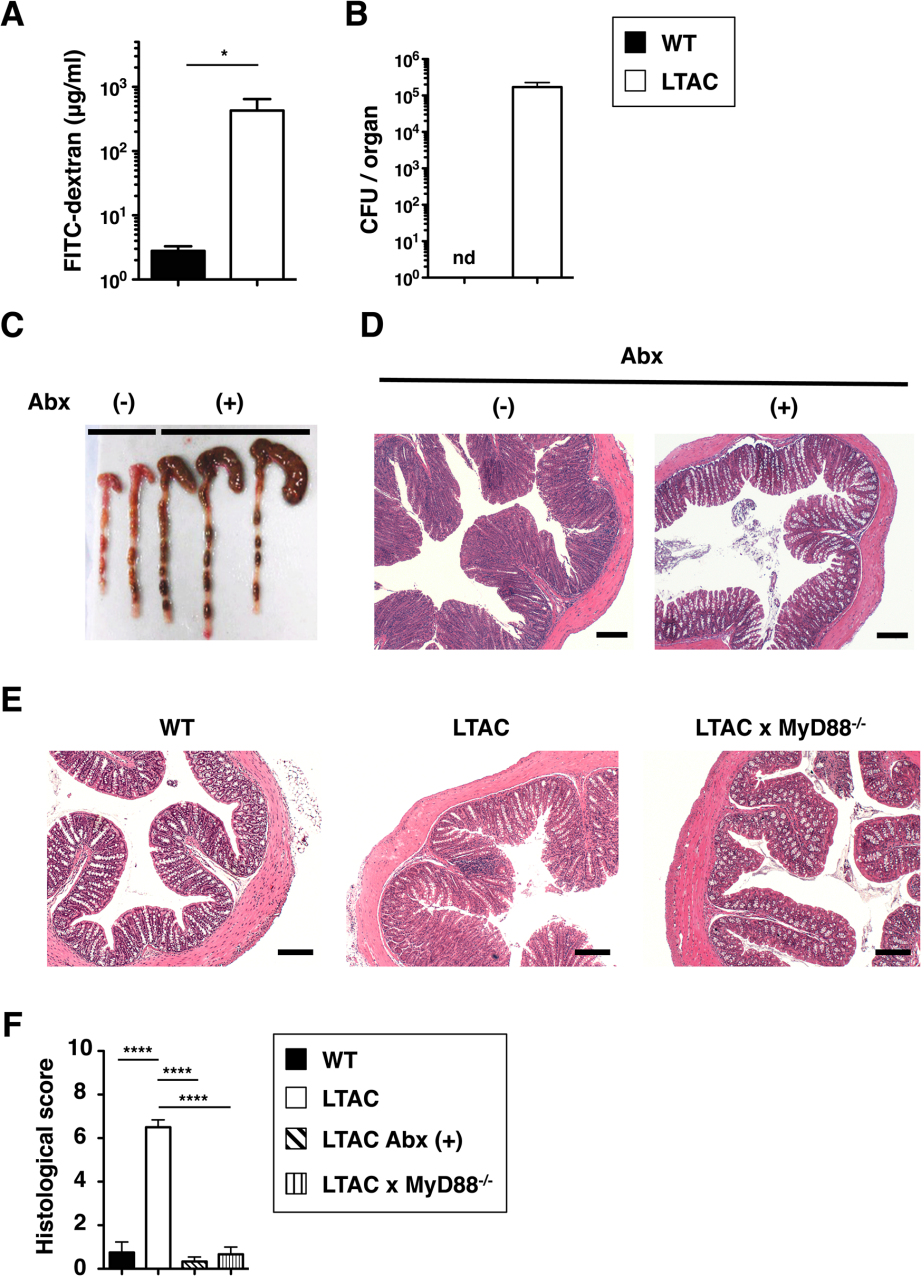
Gut microbiota and MyD88-mediated signaling promote the onset of colitis in LTAC mice. (A) Fasted mice were rectally administered with FITC-labeled dextran (2 mg / 10 g body weight) and after 3 hours blood was collected. Concentration of the FITC-dextran in the sera from WT (n = 5, filled bar) and LTAC mice (n = 7, open bar) are depicted. Data are shown as mean ± s.e.m.. (B) Bacterial titre in liver from WT (n = 5, filled bar) and LTAC mice (n = 5, open bar). Data are shown as mean ± s.e.m.. CFU, colony forming units. nd, not detected. (C and D) Six- to eight-week-old LTAC mice were subjected to intake of drinking water containing antibiotics cocktail (Abx) for 8 weeks. (C) Picture of the cecum and colon after Abx-treated (+) or left untreated (-) LTAC mice. (D) HE staining with proximal colons harvested from Abx-treated (+) or left untreated (-) LTAC mice. Data are representative of 4 to 6 individual mice. Scale bar, 200 μm. (E) HE staining with proximal colon harvested from each genotype of 12- to 16-week-old mice. Data are representative of 4 to 6 individual mice. Scale bar, 200 μm. (F) Histological score determined based upon criteria for colitis is depicted in each mice. WT (n = 4, filled bar), LTAC (equal to Abx(–)) (n = 6, open bar), LTAC Abx (+) (n = 6, hatched bar), LTAC x MyD88^**–/–**^(n = 4, vertical striped bar). Data are shown as mean ± s.e.m.. In (A), Mann-Whitney test was performed. In (B), unpaired *t* test was performed. In (F), one-way ANOVA Bonferroni’s multiple comparison test was performed. Statistical significance is indicated by *P < 0.05, ****P < 0.0001.

We further sought to identify the pathological mechanisms underlying the spontaneous colitis in LTAC mice. When tracking systemic translocation of FITC-labeled dextran from the colon, we observed that, in contrast to the absence of FITC-dextran outside of the colon in wild-type mice, massive FITC-labeled dextran was detected in the sera from LTAC mice (Fig 4A), indicative of increased intestinal epithelial permeability due to the barrier dysfunction. Such a barrier defect may permit the penetration of gut commensal bacteria into the lamina propria, followed by systemic dissemination. To check for this possibility, we cultured liver extracts for signs of bacterial translocation. In contrast to wild-type mice, large numbers of bacteria were detectable in the livers of LTAC mice (Fig 4B). These data indicated that LTAC mice were defective for gut barrier function. We next asked if depletion of bacteria in the gut could affect the disease onset. To this end, we removed the gut commensal bacteria by the treatment with a cocktail of four antibiotics. After treatment, successful elimination of enterobacteria was confirmed with enlarged ceca in LTAC mice (Fig 4C). The colons in these mice revealed no obvious symptom of colitis even at the age when untreated mice developed severe colitis (Fig 4D and 4F). As it is apparent that TLR/MyD88-mediated signaling pathway recognizes gut commensal bacteria [28], we next investigated the pathogenic role of the TLR/MyD88 pathway in promoting disease onset. We generated LTAC mice on a MyD88-deficient background and monitored these mice over 4 months after birth. Whereas all LTAC mice exhibited severe colitis, LTAC x MyD88^–/–^ mice as well as wild-type mice did not show any manifestation of inflammation (Fig 4E and 4F). Taken together, these data demonstrated that the gut microbiota-driven MyD88-mediated signaling cascade plays a pivotal role in triggering the disease in LTAC mice.

### Development of TCRαβ^+^CD8α^+^ IELs depends strictly on TAK1-mediated signaling

**Fig 5:**
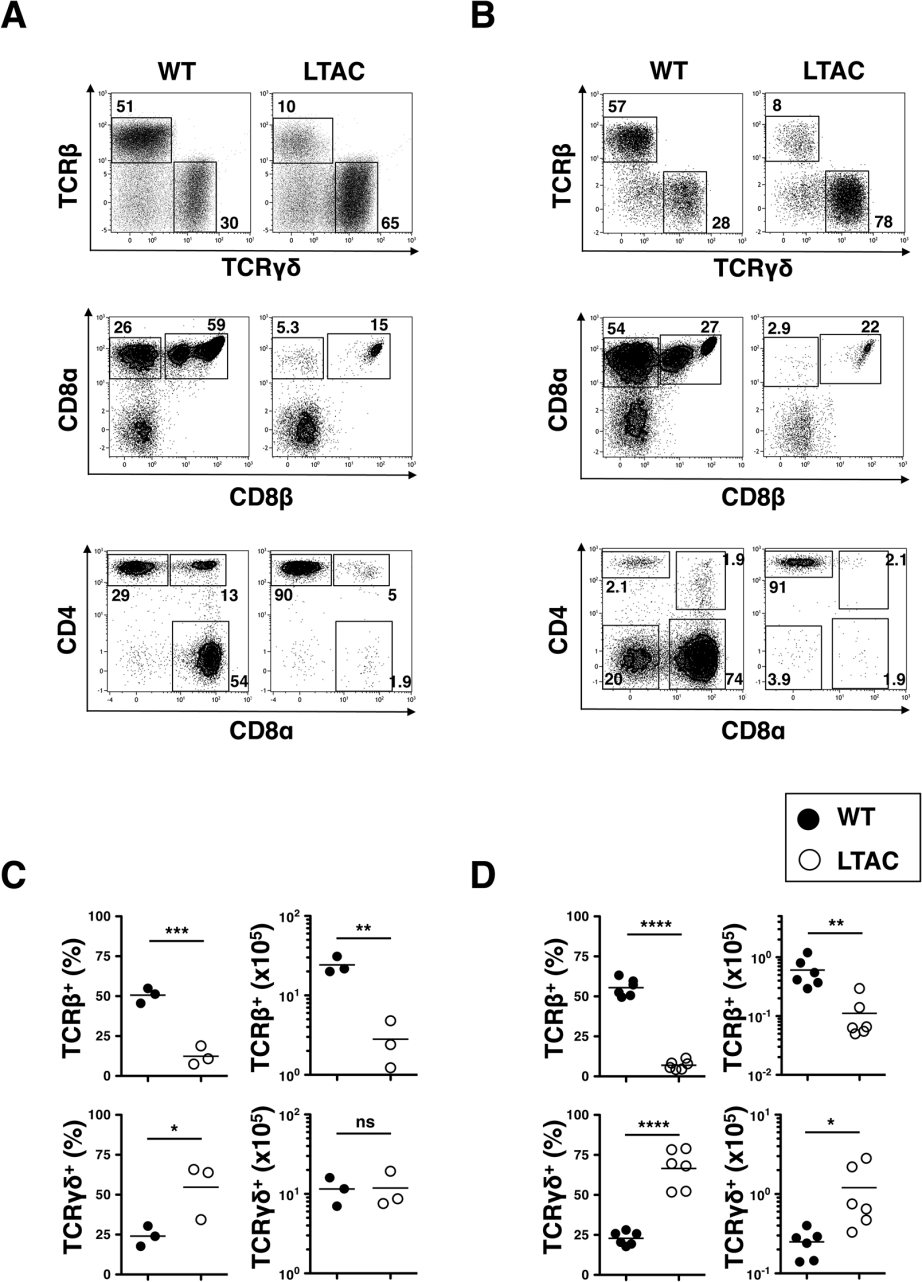
TCRαβ^+^CD8α^+^ but not TCRγδ^+^ IELs are absent in LTAC mice. (A and B) Flow cytometry of TCRβ^**+**^ and TCRγδ^**+**^ IELs in CD45^**+**^ cells (upper panels), CD8α^**+**^ and CD8β^**+**^ IELs in TCRβ^**+**^ cell population (middle panels) and CD4^**+**^ and CD8α^**+**^ IELs in TCRβ^**+**^CD8β^**–**^ cell population (lower panels) in small (A) and large (B) intestines. The plots are representative of at least three independent experiments. (C and D) Frequencies and absolute numbers of TCRβ^**+**^ or TCRγδ^**+**^ IELs in the small (C) and large (D) intestines of WT (n = 3–6, filled circle) and LTAC mice (n = 3–6, open circle), determined by flow cytometry analysis from (A) and (B), respectively. Horizontal bars represent mean. The plots are representative of at least three independent experiments. In (C) and (D), unpaired *t* tests were performed. Statistical significance was indicated by *P < 0.05, **P < 0.01, ***P < 0.001, ****P < 0.0001; ns, not significant.

We next sought to reevaluate various distinct subpopulations of intestinal immune cells in LTAC mice. Intriguingly, we found a remarkable reduction in the frequencies and absolute numbers of TCRβ^+^ IELs in both the small and large intestine of LTAC mice when compared with wild-type mice (Fig 5A and 5B; upper panels and Fig 5C and 5D). As for the subsets of TCRβ^+^ IELs, we observed that both CD8αα^+^ and CD8αβ^+^ cell populations, the major subsets in TCRαβ^+^ IELs [17], were almost completely absent in LTAC mice (Fig 5A and 5B; middle panels). Furthermore, CD4^+^CD8αα^+^ cell population in TCRβ^+^CD8β^–^ cells was significantly decreased in both the small and large intestine, and a colon exclusive TCRβ^+^CD4^–^CD8α^–^CD8β^–^ cell population was absent in LTAC mice (Fig 5A and 5B; lower panels). The remaining subtle TCRβ^+^ IEL fraction detected in both the small and large intestine of LTAC mice was CD4^+^ T cells (Fig 4A and 4B; lower panels). In contrast, we observed a marked increase in the frequency of TCRγδ^+^ IELs in both the small and large intestine of LTAC mice, and the absolute numbers of TCRγδ^+^ IELs were also significantly increased in the colon but not small intestine of LTAC mice (Fig 5A and 5B; upper panels and Fig 5C and 5D). These results, along with the situation in lamina propria, indicated that in the gut of LTAC mice TCRβ^+^CD8α^+^ IELs were almost completely absent possibly due to TAK1 deficiency, whereas the remaining T cells, largely CD4^+^ and γδ^+^ T cells, retained a sufficient TAK1 protein.

**Fig 6:**
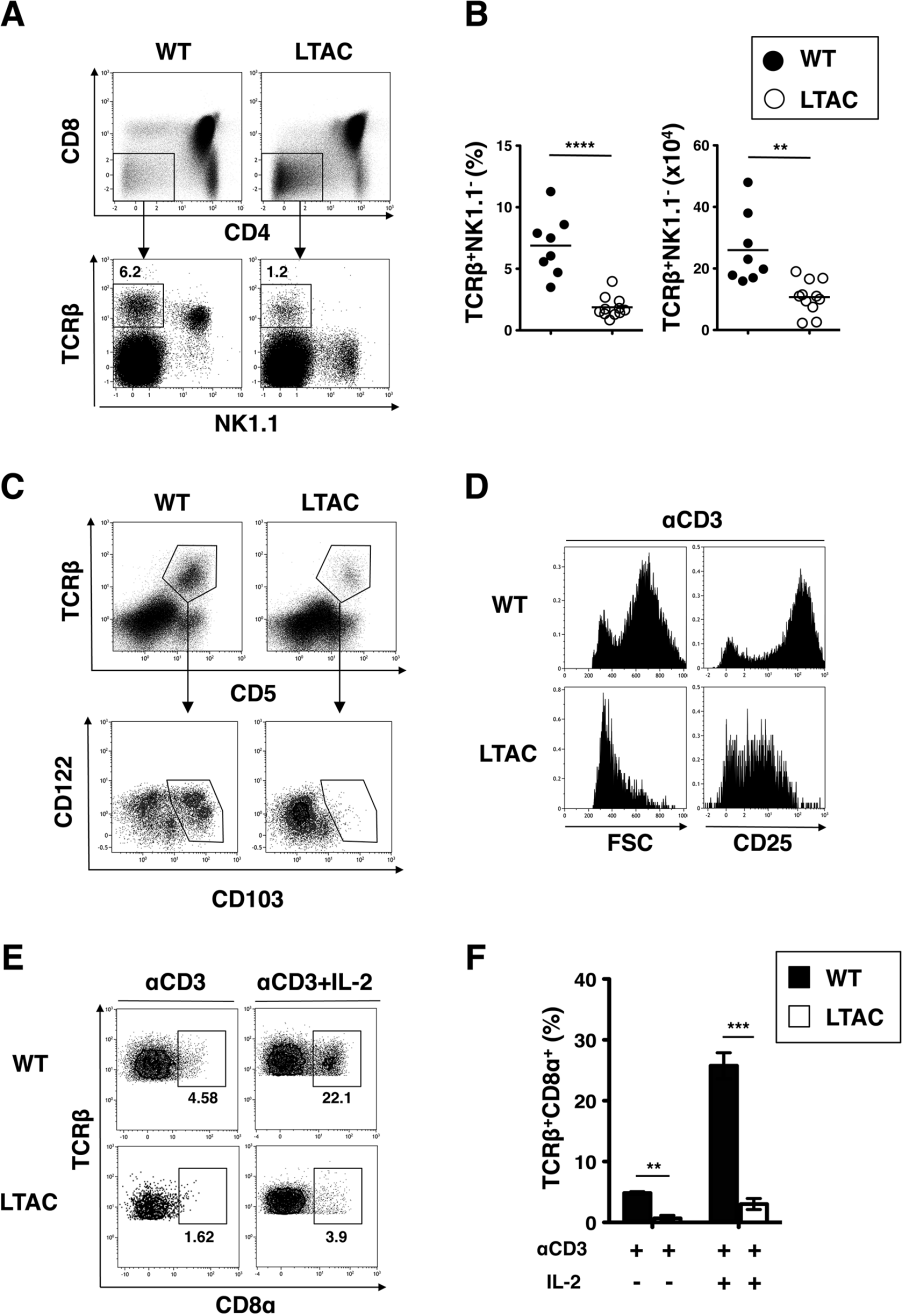
TAK1-dependent TCR-driven signaling pathway is essential for the development of TCRαβ^+^ IELs. (A) Flow cytometry of TCRβ^**+**^CD4^**–**^CD8^**–**^NK1.1^**–**^ precursor cells in thymus. The plots are representative of at least three independent experiments. (B) Frequency (left) and absolute number (right) of TCRβ^**+**^ after gating out of CD4^**+**^, CD8^**+**^ and NK1.1^**+**^ cells of WT (n = 8, filled circle) and LTAC mice (n = 9, open circle), determined by flow cytometry analysis from (A). Horizontal bars represent mean. (C) Characterization of cell surface proteins on TCRβ^**+**^ thymic precursor cells after depletion of CD4^**+**^, CD8^**+**^ and NK1.1^**+**^ cells of WT and LTAC mice. The plots are representative of at least three independent experiments. (D, E and F) Thymocytes after depletion of CD4^**+**^, CD8^**+**^ and NK1.1^**+**^ cells were cultured with the plate-bound anti-CD3 antibody (αCD3) in the absence or presence of IL-2 for 3 days. In (D), forward scatter (FSC) for monitoring cell size and inducible expression of CD25 are depicted as histograms. In (E), inducible expression of CD8α on TCRβ^**+**^ precursor cells is shown. The plots are representative of at least three independent experiments. In (F), frequency of TCRβ^**+**^CD8α^**+**^ cells in cultured cells from WT (n = 3, filled bar) and LTAC mice (n = 3, open bar) are shown. Data are shown as mean ± s.e.m.. In (B) and (F), unpaired *t* tests were performed. Statistical significance was indicated by **P < 0.01, ***P < 0.001, ****P < 0.0001.

We next sought to determine at which stage the IEL development was impaired in LTAC mice. Since it was shown that IELs developed from thymic precursors [29], we examined thymocytes isolated from LTAC mice for IEL precursors. Flow cytometry analysis showed that thymic IEL precursors, defined as TCRαβ^+^CD4^–^CD8^–^NK1.1^–^, were significantly decreased in both the frequency and absolute number in LTAC mice when compared with wild-type mice (Fig 6A and 6B). Detailed analysis further demonstrated that such a reduction could be explained by the almost complete loss of CD103-positive cell population coexpressing both CD5 and CD122 (Fig 6C). Additionally, we found that TCRβ^+^NK1.1^+^ natural killer T (NKT) cell population in the thymus and liver and α-GalCer/CD1d-reacted NKT cell population in the liver were hardly detectable in LTAC mice (Fig 6A and S2 Fig), indicating that the development of NKT cells is also defective in the absence of TAK1.

The discovery of the decreased thymic IEL precursors in LTAC mice prompted us to specify signaling pathway(s) in which TAK1 functions. Since TAK1 is already known as an important component for TCR-mediated signaling pathway in thymocytes, we investigated cellular responsiveness of IEL precursors to TCR stimulation in an *in vitro* cell culture system. When TCRαβ^+^CD4^–^CD8^–^NK1.1^–^ cells from the thymus were stimulated with an agonistic anti-CD3 antibody, cells from wild-type mice showed an enlarged cell size and a robustly induced expression of CD25, IL-2Rα chain, but such changes were scarcely observed in those from LTAC mice (Fig 6D). Moreover, when stimulated only with the agonistic anti-CD3 antibody, CD8α expression was induced on a fraction of TCRβ^+^CD4^–^CD8^–^NK1.1^–^ precursor cells from wild-type mice. However, in the presence of IL-2 plus anti-CD3 antibody elevated CD8α expression was observed (Fig 6E and 6F). In addition, the TCRβ^+^CD8α^+^ fraction could be subdivided further; the major CD8α^+^ and minor CD8α^+^CD8β^+^ and CD8α^+^CD4^+^ populations (S3 Fig). In contrast, the precursors from LTAC mice treated with the agonistic anti-CD3 antibody showed inefficient induction of CD8α regardless of whether IL-2 was present (Fig 6E and 6F and S3 Fig). This poor CD8α induction on LTAC-derived precursors seemed to be due to the failure of TCR stimulation to up-regulate CD25 (Fig 6D) as well as to directly induce CD8α (Fig 6E and 6F). Taken together, these data revealed that TAK1-dependent TCR signaling was crucial for the early development of IELs.

### Exogenous TCRαβ^+^CD8α^+^ IELs ameliorate colitis in LTAC mice

**Fig 7:**
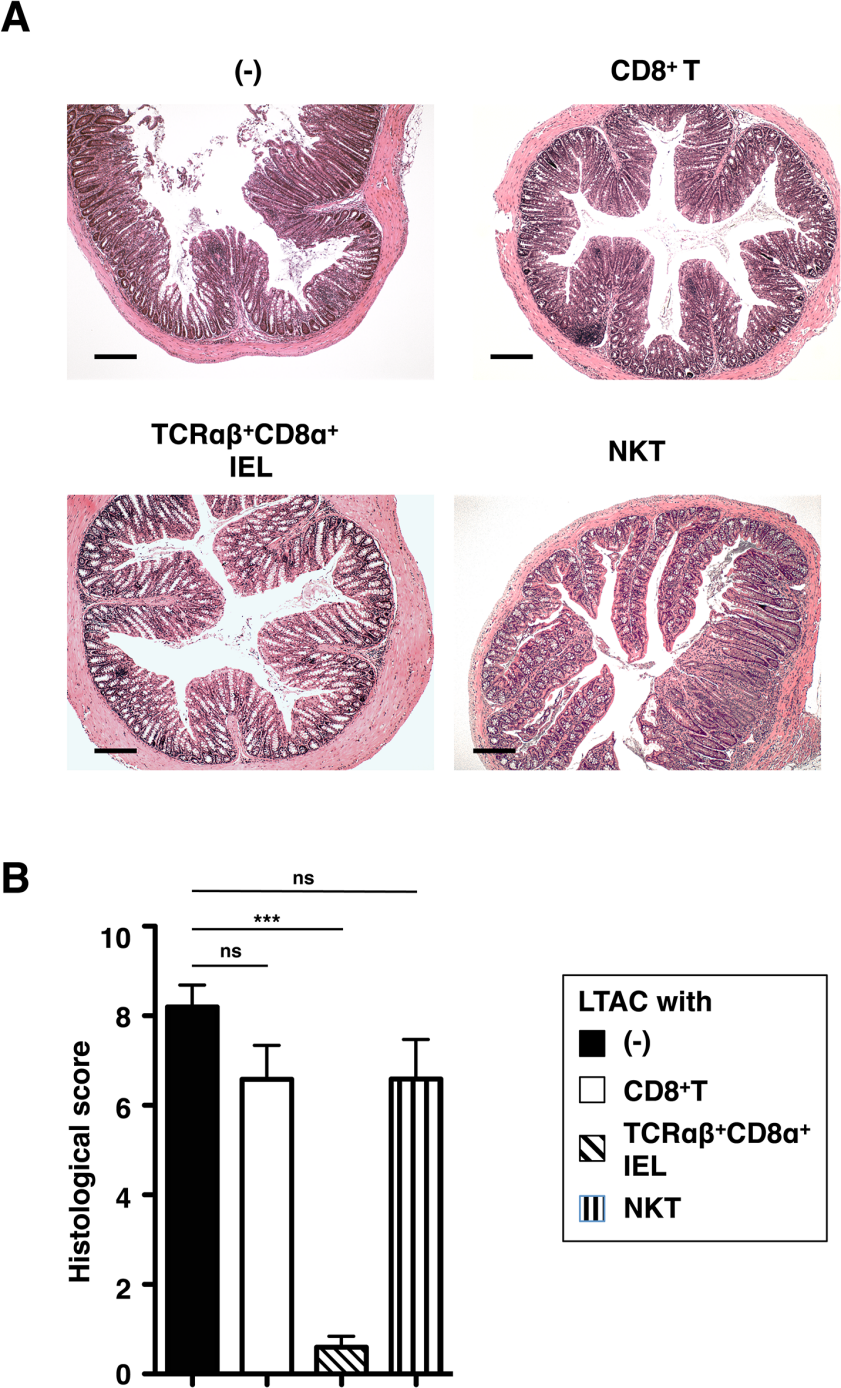
Administration of TCRαβ^+^CD8α^+^ IELs ameliorates spontaneous colitis in LTAC mice. Individual T cell subsets sorted from the spleen or the small and large intestine of CD45.1^**+**^ C57BL/6 mice were intravenously transferred into 6- to 8-week-old LTAC mice. Eight weeks after transfer, the mice were used for analysis. (A) HE staining with proximal colons. Data are representative of 5 to 6 individual mice. Scale bar, 200 μm. (B) Histological score determined based upon criteria for colitis is depicted in each mouse group. No transfer (–) (n = 5, filled bar), CD8^**+**^ T (n = 6, open bar), TCRαβ^**+**^CD8α^**+**^ IEL (n = 6, hatched bar), NKT cells (n = 6, vertical striped bar). Data are shown as mean ± standard error of the mean (s.e.m.). In (B), one-way analysis of variance (ANOVA) Bonferroni’s multiple comparison test was performed. Statistical significance is indicated by ***P < 0.001; ns, not significant.

In order to further explore the relationship between the reduction of T lymphocytes and spontaneous colitis, we considered that deficiency of T cell subset(s) in LTAC mice might have an important cue to prevent the disease onset. To this end, we transferred individual T cell subsets purified from CD45.1^+^ C57BL/6 mice into 6- to 8-week-old LTAC mice that had not yet displayed any signs of the colitis and examined these mice for colitis 8 weeks after transfer. We observed inflammatory infiltrates and massive tissue damages, the typical features for colitis, in LTAC mice transferred with conventional CD8^+^ T or NKT cells as well as in untreated LTAC mice (Fig 7A and 7B). Surprisingly, when TCRαβ^+^CD8α^+^ IELs were transferred into LTAC mice, we noticed a significant amelioration of colitis (Fig 7A and 7B). Collectively, these data indicated that the lack of TCRαβ^+^CD8α^+^ IEL in the intestinal epithelial layer led to the pathogenesis of colitis in LTAC mice.

## Discussion

In the present study, we described a detailed characterization of LTAC mice, which have been identified by chance to manifest spontaneous colitis in Lck-cre:TAK1^fl/fl^ mice. Overall, multiple events were involved in provoking local inflammation in the colon. One of these events was, despite the systemic T lymphopenia, an unanticipated accumulation of aberrant CD4^+^ T cells including regulatory T cells retaining TAK1 in mucosal tissues of LTAC mice, eventually contributing to the disease onset. Additionally, the gut commensal bacteria and evoking the MyD88-dependent signaling pathway were essential for the initiation and persistence of inflammation in the colon. We found that LTAC mice were devoid of TCRαβ^+^CD8α^+^ IELs in both the small and large intestine, reflecting the impairment in early IEL development caused presumably by a poor TCR signaling in the absence of TAK1 in thymic IEL precursors. The most important finding in this study is the ability of TCRαβ^+^CD8α^+^ IELs to suppress the onset of colitis when transferred into LTAC mice. A plausible scenario would be that IELs prevent colitis development through maintaining mucosal barrier function limiting the burden of disseminated enterobacteria into peripheral organs in LTAC mice. Based on these interesting features, we propose that LTAC mice could be a unique animal model useful to explore the regulatory function of TCRαβ^+^CD8α^+^ IELs in mucosal immune homeostasis.

Considering the immunopathological mechanism of colitic LTAC mice, there is no doubt that appearance and accumulation of aberrant T cells contribute to the onset of colitis. Of note, the fact that suppressive activity was decreased in leaky regulatory T cells in LTAC mice gained a better understanding of the etiology. Although the mechanism from which such the dysfunctional regulatory T cells arise remains to be explicit, previous reports that described the plasticity of regulatory T cells in lymphopenic or inflammatory conditions may provide clues to consider what happens in regulatory T cells in LTAC mice [30,31]. In addition, involvement of gut commensal bacteria as detrimental factors in LTAC mice is not negligible when considering the etiology. It is still controversial as to how the gut microbiota affects induction of abnormal T cell activation in LTAC mice, whereas the finding that gut homing receptors such as α4β7, CCR9 and CD103 were up-regulated in leaky CD4^+^ T cells in the mLNs of LTAC mice allows us to conceive of the participation of dendritic cells (DCs) resided in mucosal sites to modulate T cell phenotype. For instance, it is now appreciated that retinoic acid (RA), a metabolite of vitamin A produced from gut-associated cells including LPDCs, plays an essential role for induction of the gut homing receptors [32]. However, we did not observe any significant differences in the frequency of recruited LPDCs from the gut and expressions of the RA synthases RALDH1/2 and TGFβ in the mLNs between wild-type and LTAC mice (S4 Fig), suggesting that the DCs themselves are unlikely to be functionally problematic. Meanwhile, we are not able to exclude a possibility that continuous exposure of LPDCs to gut commensal bacteria ensures the persistence of antigen presentation to T cells in the colon of LTAC mice.

We found that IEL development was impaired in LTAC mice at the level of thymic precursors. Our analysis also demonstrated that TAK1-dependent TCR signaling was required for IEL development, providing a plausible mechanistic insight that both TCR- and IL-2R-mediated signals have a synergistic impact on differentiation of thymic precursors to TCRαβ^+^CD8αα^+^ IELs. Given that TAK1 in thymocytes is essential for the TCR-mediated NF-κB activation and CD25 is known as a target gene for NF-κB [24–26,33], the TAK1-dependent TCR signaling via NF-κB activation is thought to be a critical step for progression of IEL development in thymus. On the other hand, recent reports have shown that IL-15 signaling and a transcription factor T-bet play crucial roles for IEL development [34,35]. However, it should be noted that mice deficient for IL-15 or T-bet still possess substantial frequencies and numbers of thymic IEL precursors. This finding indicates that unlike TAK1 these molecules are most likely critical for homeostatic regulation of IELs in intestine rather than thymus. Additionally, agonist selection, a phenomenon wherein unconventional T cells such as IELs, regulatory T cells and NKT cells are thought to experience strong interaction of TCR with self-ligands in the thymus, is known as an important step toward terminal differentiation of each cell lineage [36]. Given that besides IELs, LTAC mice exhibited a developmental defect of regulatory T cells and NKT cells due to TAK1 deficiency in T cells, our results indicate that a TAK1-dependent TCR signaling event could be an early critical step to achieve agonist selection in the thymus, followed by maturation of the unconventional T cells.

From the point of view of amelioration of the disease, it is remarkable that transfer of TCRαβ^+^CD8α^+^ IELs restrained spontaneous colitis, consistent with previous reports that the cells have an ability to suppress gut inflammation [19,20,22,34]. At present, although there is no direct evidence to explain how the IELs control the homeostasis in the gut, the following notions may be applicable. One is that IELs have a significant impact on modulation of the intestinal barrier function in collaboration with epithelial cells for the inhibition of penetration of enterobacteria, as exemplified by a recent report [37]. Another is that IELs may have a potential immune regulatory function as previously described [15–17]. For example, a report pointed out that the TCRαβ^+^CD8αα^+^ IELs had a characteristic feature that implied immune regulation from a microarray analysis [38]. Additionally, we observed that LTAC mice had considerable frequency and number of TCRγδ^+^ cells, which possess TAK1. Whereas previous reports demonstrated that TCRγδ^+^ IELs contributed to a resolution of inflammatory lesions in the intestines [18,21], the fact that LTAC mice develop colitis even in the presence of TCRγδ^+^ IELs may support the importance of TCRαβ^+^CD8αα^+^ IELs to suppress abnormal immune response in the gut. Collectively, our current study emphasized the emerging role of TAK1 in configuring the gut-specialized T cell subset and defined LTAC mice as a beneficial animal model of IBD. In addition, these mice could prove instrumental in evaluating IEL function to control immune homeostasis in the gut.

## Supporting Information

S1 Fig Cytokine expression of CD4^+^ T cells in mLN. (A and B) Flow cytometry of intracellular cytokines in TCRβ^+^CD4^+^ cells from mLN and frequencies of each subset of cytokine-producing cells in TCRβ^+^CD4^+^ cells. The plots are representative of four independent experiments. WT (n = 4, filled circle) and LTAC mice (n = 4, open circle), calculated by flow cytometry analysis. Horizontal bars represent mean. In (B), unpaired *t* tests were performed. Statistical significance was indicated by **P < 0.01, ****P < 0.0001.
(TIF)

S2 Fig NKT cells are absent in LTAC mice. (A) Flow cytometry of liver NKT cells. The plots are representative of three independent experiments. (B) Frequency and absolute number of TCRβ^+^αGalCer^+^ NKT cells in the liver of WT (n = 3, filled circle) and LTAC mice (n = 3, open circle), determined by flow cytometry analysis from (A). Horizontal bars represent mean. In (B), unpaired *t* tests were performed. Statistical significance was indicated by **P < 0.01.
(TIF)

S3 Fig Characterization of TCRβ^+^ precursor cells for IELs by *in vitro* cell culture. Thymocytes after depletion of CD4^+^, CD8^+^ and NK1.1^+^ cells were cultured with the plate-bound anti-CD3 antibody in the presence of IL-2 for 3 days. Inducible expressions of CD8α, CD8β and CD4 in TCRβ^+^ cell population are depicted. The plots are representative of three independent experiments.
(TIF)

S4 Fig Comparison of DC subpopulations and expression of RALDH1, 2 and TGFβ in mLN. Flow cytometry analysis with CD45^+^ cells in the mLN of 8- to 12-week-old WT and LTAC mice. (A) Cell surface staining for detection of the migratory DC subpopulations in CD11c^+^I-Ab^hi^. The plots are representative of three independent experiments. (B) Frequencies of each DC subtype in CD11c^+^I-Ab^hi^ cells in the mLNs of wild type (n = 3, filled bar) and LTAC mice (n = 3, open bar), determined by flow cytometry analysis from (A). Data are shown as mean ± s.e.m.. (C) Real time PCR was performed using total RNAs from the mLNs of 8- to 12-week-old WT and LTAC mice. Data are representative of three independent experiments (mean ± s.d.).
(TIF)
