# Supplementary figures and images for: Conditional Deletion of TAK1 in T Cells Reveals a Pivotal Role of TCRαβ+ Intraepithelial Lymphocytes in Preventing Lymphopenia-Associated Colitis
Source: PLoS One. 2015 Jul 1;10(7):e0128761. doi: 10.1371/journal.pone.0128761 (PMC4489433; doi:10.1371/journal.pone.0128761)

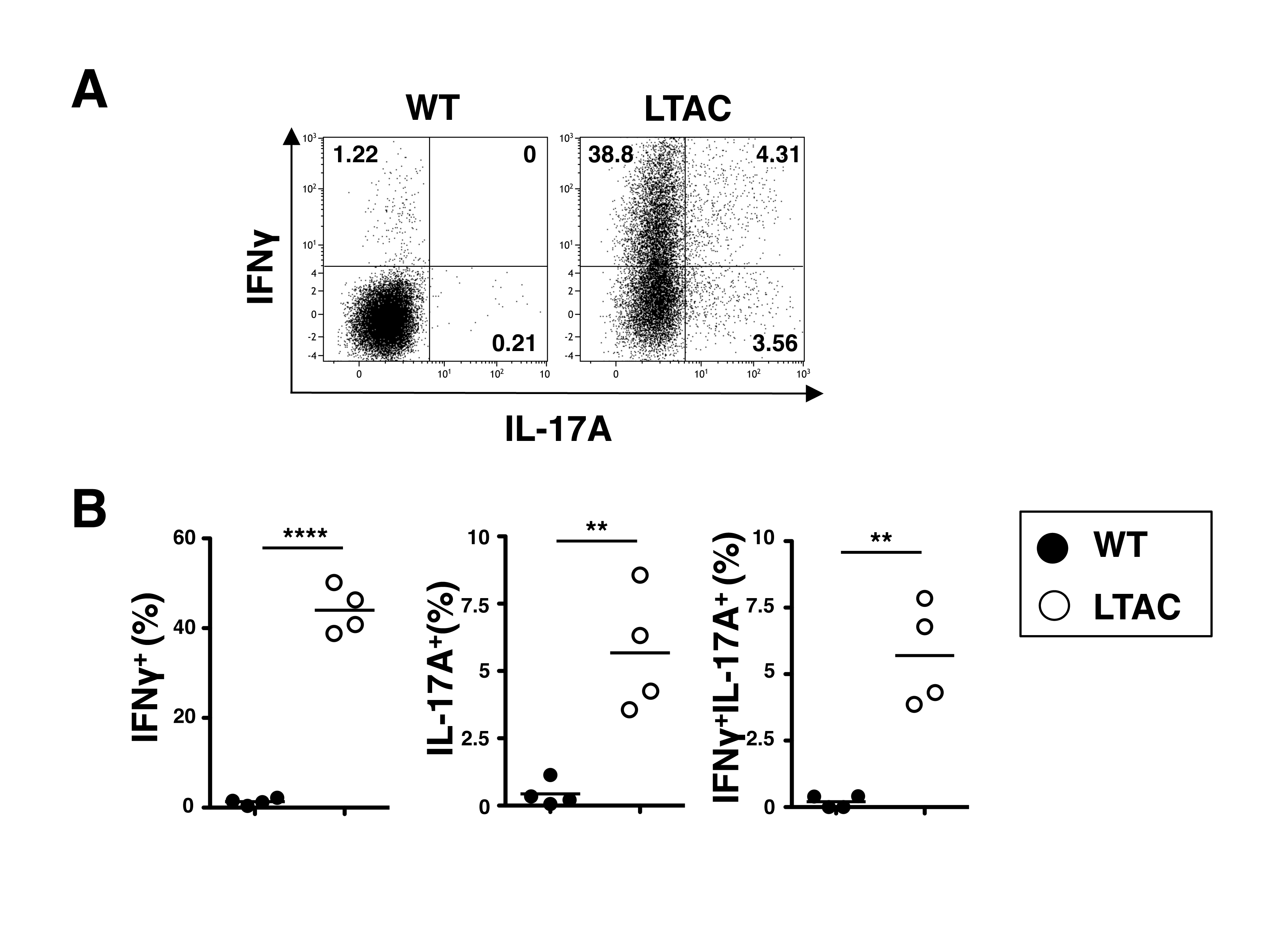

Supplement: S1 Fig — (A and B) Flow cytometry of intracellular cytokines in TCRβ+CD4+ cells from mLN and frequencies of each subset of cytokine-producing cells in TCRβ+CD4+ cells. The plots are representative of four independent experiments. WT (n = 4, filled circle) and LTAC mice (n = 4, open circle), calculated by flow cytometry analysis. Horizontal bars represent mean. In (B), unpaired t tests were performed. Statistical significance was indicated by **P < 0.01, ****P < 0.0001. (TIF) [file pone.0128761.s001.tif]

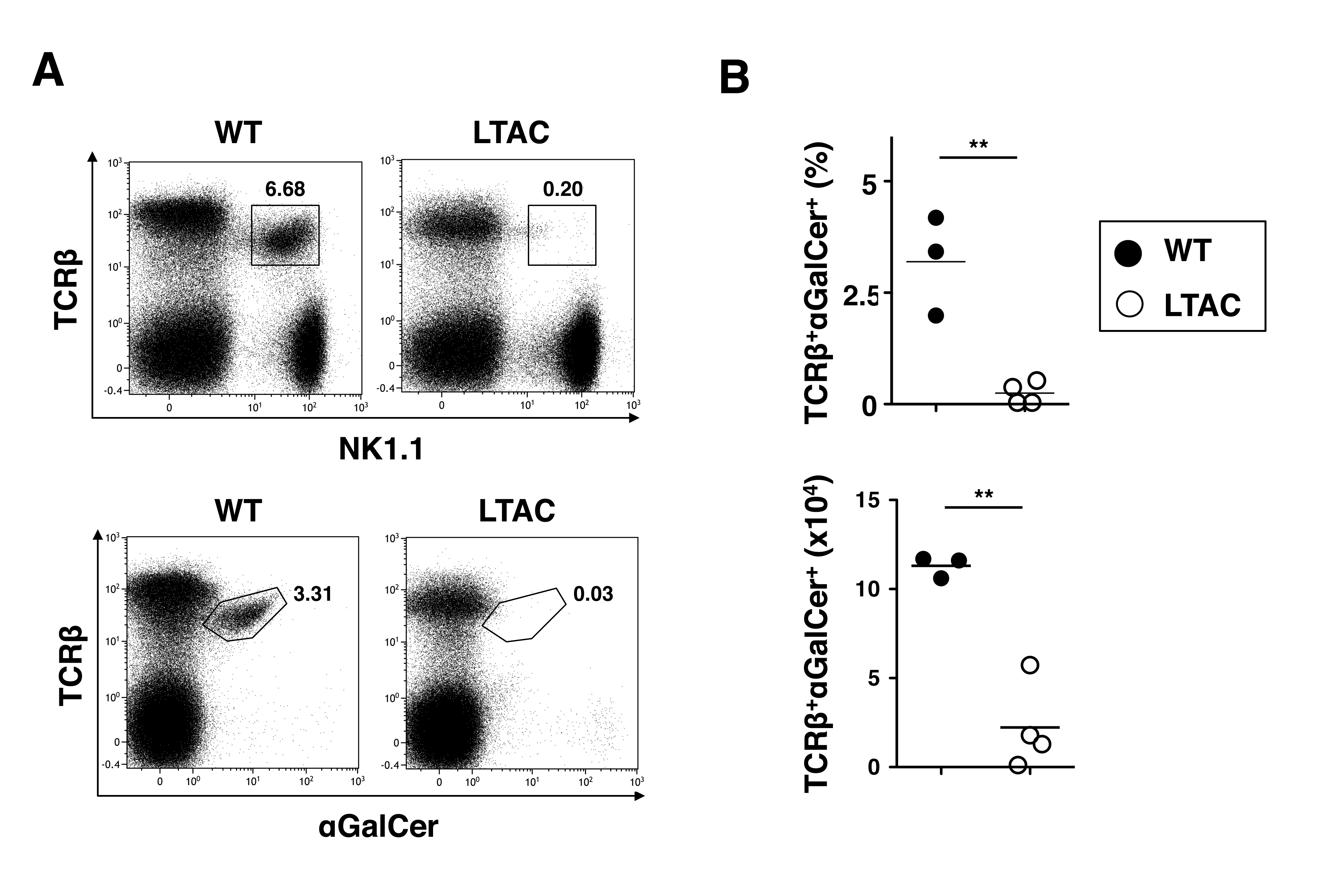

Supplement: S2 Fig — (A) Flow cytometry of liver NKT cells. The plots are representative of three independent experiments. (B) Frequency and absolute number of TCRβ+αGalCer+ NKT cells in the liver of WT (n = 3, filled circle) and LTAC mice (n = 3, open circle), determined by flow cytometry analysis from (A). Horizontal bars represent mean. In (B), unpaired t tests were performed. Statistical significance was indicated by **P < 0.01. (TIF) [file pone.0128761.s002.tif]

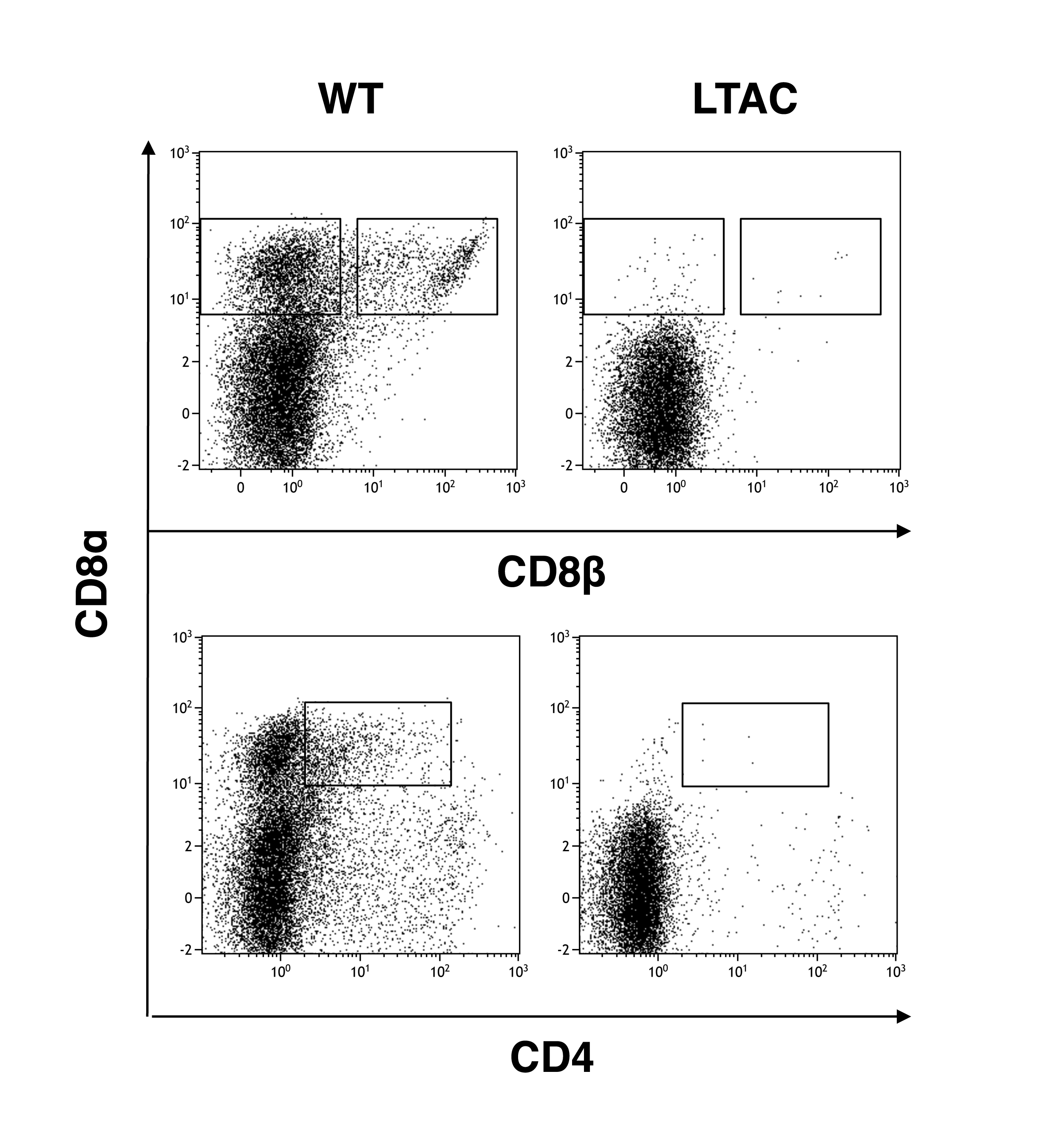

Supplement: S3 Fig — Thymocytes after depletion of CD4+, CD8+ and NK1.1+ cells were cultured with the plate-bound anti-CD3 antibody in the presence of IL-2 for 3 days. Inducible expressions of CD8α, CD8β and CD4 in TCRβ+ cell population are depicted. The plots are representative of three independent experiments. (TIF) [file pone.0128761.s003.tif]

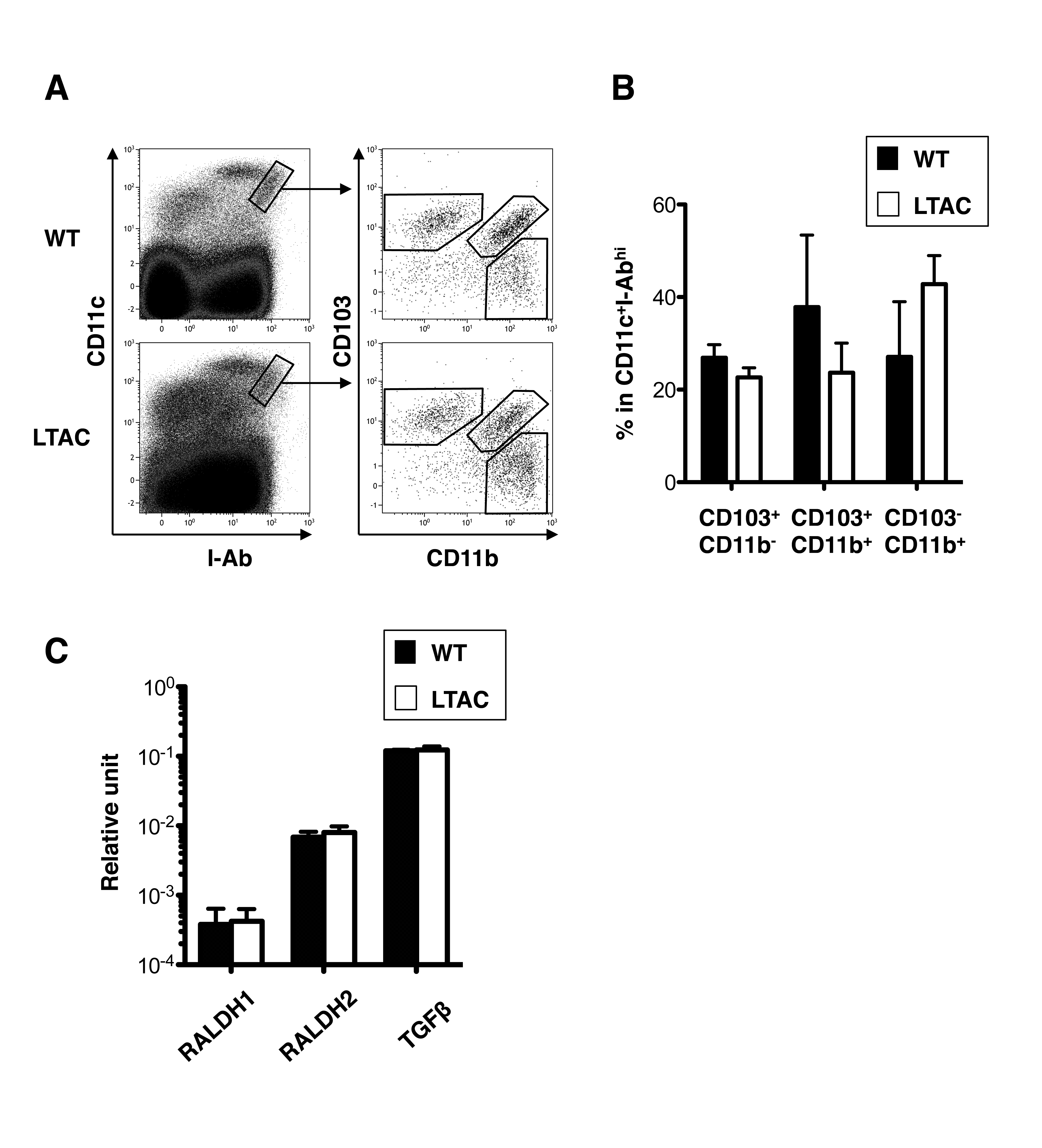

Supplement: S4 Fig — Flow cytometry analysis with CD45+ cells in the mLN of 8- to 12-week-old WT and LTAC mice. (A) Cell surface staining for detection of the migratory DC subpopulations in CD11c+I-Abhi. The plots are representative of three independent experiments. (B) Frequencies of each DC subtype in CD11c+I-Abhi cells in the mLNs of wild type (n = 3, filled bar) and LTAC mice (n = 3, open bar), determined by flow cytometry analysis from (A). Data are shown as mean ± s.e.m.. (C) Real time PCR was performed using total RNAs from the mLNs of 8- to 12-week-old WT and LTAC mice. Data are representative of three independent experiments (mean ± s.d.). (TIF) [file pone.0128761.s004.tif]
